# Effectiveness and safety of tumor-treating fields therapy for glioblastoma: A single-center study in a Chinese cohort

**DOI:** 10.3389/fneur.2022.1042888

**Published:** 2023-01-09

**Authors:** Lei She, Xuan Gong, Lin Su, Chao Liu

**Affiliations:** ^1^Department of Oncology, National Clinical Research Center for Geriatric Disorders, Xiangya Hospital, Central South University, Changsha, Hunan, China; ^2^Hunan Key Laboratory of Pharmacogenetics, Department of Clinical Pharmacology, Xiangya Hospital, Central South University, Changsha, Hunan, China; ^3^Department of Neurosurgery, Xiangya Hospital, Central South University, Changsha, Hunan, China

**Keywords:** newly diagnosed glioblastoma, recurrent glioblastoma, tumor-treating fields, survival benefit, adverse events

## Abstract

**Objective:**

Tumor-treating fields (TTFields) are a new therapeutic modality for patients with glioblastoma (GBM). However, studies on survival outcomes of TTFields are rarely reported in China. This study aimed to examine the clinical efficacy and safety of TTFields therapy for GBM in China.

**Methods:**

A total of 93 patients with newly diagnosed GBM (ndGBM) and recurrent GBM (rGBM) were included in our study retrospectively. They were divided into two groups based on whether they used TTFields. Progression-free survival (PFS), overall survival (OS), and toxicities were assessed.

**Results:**

Among the patients with ndGBM, there were 13 cases with TTFields and 39 cases with no TTFields. The median PFS was 15.3 [95% confidence interval (CI): 6.5–24.1] months and 10.6 (95% CI: 5.4–15.8) months in the two groups, respectively, with *P* = 0.041. The median OS was 24.8 (95% CI: 6.8–42.8) months and 18.6 (95% CI: 11.4–25.8) months, respectively, with *P* = 0.368. Patients with subtotal resection (STR) who used TTFields had a better PFS than those who did not (*P* = 0.003). Among the patients with rGBM, there were 13 cases with TTFields and 28 cases with no TTFields. The median PFS in the two groups was 8.4 (95% CI: 1.7–15.2) months and 8.0 (95% CI: 5.8–10.2) months in the two groups, respectively, with *P* = 0.265. The median OS was 10.6 (95% CI: 4.8–16.4) months and 13.3 (95% CI: 11.0–15.6) months, respectively, with *P* = 0.655. A total of 21 patients (21/26, 80.8%) with TTFields developed dermatological adverse events (dAEs). All the dAEs could be resolved or controlled.

**Conclusion:**

TTFields therapy is a safe and effective treatment for ndGBM, especially in patients with STR. However, it may not improve survival in patients with rGBM.

## 1. Introduction

Glioblastoma (GBM) is the most common primary malignant intracranial tumor, with characteristics of remarkably high heterogeneity, strong invasiveness, and poor outcomes (1, 2). Currently, the standard therapy for newly diagnosed GBM (ndGBM) involves maximal safe resection followed by concurrent radiotherapy and temozolomide (TMZ) administration (3). However, such therapy only shows a median progression-free survival (PFS) of 6.9 months [95% confidence interval (CI): 5.8–8.2] and median overall survival (OS) of 14.6 (95% CI: 13.2–16.8) months (4). To improve the survival outcomes for GBM, clinical trials for targeted therapy, immunotherapy, and a combination of TMZ with other chemotherapeutics have been extensively evaluated, and most of them are phase I/II clinical trials (5–7). Only a few phase III clinical trials have been reported for the ndGBM population (8–10). Despite the standardized treatment, ~85% of GBM cases relapse within 2 years (11, 12). The outcome in patients with recurrent GBM (rGBM) is even worse, with a median OS of ~6 months (13). Currently, there is no category 1 recommendation for the treatment of rGBM, and the majority of patients receiving comprehensive treatment experience a decline in their quality of life, including neurocognitive and physical functions (14, 15).

Tumor-treating fields (TTFields) therapy provides low-intensity, intermediate frequency, and alternating electric fields. The mechanism of action underlies interference with the mitosis of cancer cells through the action of microtubulins, eventually suppressing cancer cell growth (16, 17). In a previous phase III clinical trial (EF-11) on rGBM, TTFields therapy did not show any remarkable improvement in median OS (6.6 vs. 6.0 months; *P* = 0.27) or PFS (2.2 vs. 2.1 months; *P* = 0.16) as compared to chemotherapy, whereas it was superior in improving the quality of life of patients owing to fewer severe adverse events (AEs) (6 vs. 16%; *P* = 0.022) (18). Given these positive results, the TTFields therapy was officially adopted in the National Comprehensive Cancer Network (NCCN) guidelines in 2013 for the treatment of rGBM. The Patient Registry Dataset (PRiDe) study reported that the TTFields therapy contributed to the 1-year survival of 44% in patients with rGBM (19). In the subsequent phase III clinical trial (EF-14) on patients with ndGBM, the combination of TTFields therapy with TMZ was found to be superior to TMZ alone, with both higher median PFS (6.7 vs. 4.0 months; *P* < 0.001) and OS (20.6 vs. 16.0 months; *P* < 0.001). The combination strategy did not increase the incidence of AEs (44 vs. 48%; *P* = 0.58) (20, 21). In 2019, the NCCN guidelines recommended the Stupp regimen plus TTFields therapy as the category 1 treatment for ndGBM and the TTFields therapy as the category 2B treatment for rGBM (22), in accordance with the *Chinese Standard Diagnosis and Treatment for Glioma, 2018*.

Clinical studies about TTFields, including EF-11, EF-14, and PRiDe, have been mostly performed in European and American populations, except for the 39 Korean patients included in EF-14. However, studies on survival outcomes of TTFields are rarely reported in China. This study aimed to examine the clinical efficacy and safety of TTFields therapy for GBM in a retrospective cohort in China.

## 2. Methods

### 2.1. Patients selection

Between January 2013 and May 2021, data from 93 patients were evaluated retrospectively at the Xiangya Hospital of Central South University. Patients with ndGBM and rGBM eligible for this study were 18 years or older, with a Karnofsky performance score (KPS) of 50 or higher, and histologically, the pathology was confirmed as supratentorial glioblastoma. All participants had undergone the safest debulking surgery possible, followed by concurrent chemoradiotherapy. Patients with implanted electronic medical devices, as well as those with other malignant tumors or serious diseases, were excluded from our study. They were divided into groups with TTFields group and without TTFields group based on whether they used TTFields.

### 2.2. Treatment strategy

Patients with ndGBM received surgery (maximum tumor resection with safety), intensity-modulated radiotherapy (IMRT) (2.0 Gy/day, 5 days a week for 60 Gy), temozolomide (TMZ) concurrent chemotherapy (75 mg/m^2^/day), and TMZ adjuvant chemotherapy (AC) (150 mg/m^2^/day in the first cycle, 200 mg/m^2^/day from the second cycle). Patients in the TTFields group got extra electric field treatment during AC.

Patients with rGBM were treated with TMZ chemotherapy or targeted treatment (nimotuzumab, bevacizumab, or anlotinib). Electric field treatment was used on patients in the TTFields group in addition to chemotherapy and targeted therapy.

All patients treated with electric field underwent examinations for full-length of the 68 genes most related to glioma. The specific process of TTFields was as follows. The NovaTTFields-200A device (Novocure, Israel) was used. Low-intensity (2 V/cm), intermediate frequency (200 kHz), and alternating electric fields were placed at the tumor regions. Two pairs of electric field patches were attached to the scalp surface of patients. Specific procedures of the TTFields therapy were in four steps. (1) Before initiating the therapy, the general conditions and indications of patients were assessed. Written informed consent was obtained from all patients. (2) Patients were asked to provide the latest head magnetic resonance imaging (MRI) scan data (within the last 1 month), and learning the use of the NovaTTFields-200A device was aided by the specialists from the Novocure company. Head size and MRI scan data were combined to determine the best patch position, and patients were guided to place the patches. (3) After patch placement, regular follow-up was performed to observe toxicity, general conditions, and provide treatment for symptomatic individuals. The MRI scan was required every 2 months or on suspicion of tumor progression. The RANO criteria were used to assess the therapeutic efficacy. Patients were encouraged to have the patches placed for more than 18 h each day. (4) Compliance report was generated every month with the support of the NovaTTFields-200A device and subsequently sent to doctors. The contents included the average daily use of the device and the overall compliance data of patients during the treatment period.

### 2.3. Evaluation of the therapeutic efficacy and toxicity

MRI scans were examined every 2 months or on suspicion of tumor progression. The disease progression was accessed every 2 months after radiotherapy according to the Response Assessment in Neuro-Oncology (RANO) criteria. Progression in the radiation field within 3 months (12 weeks) after the completion of chemoradiotherapy was needed to observe carefully to differentiate from pseudoprogression. Regular follow-up visits were performed until disease progression or death. According to Common Terminology Criteria for Adverse Events, v5.0 (CTCAE v5.0) and TTFields dermatological adverse events (dAEs) criteria, the toxicity in each patient was evaluated. Scalp examination was performed every 2 weeks after the removal of the sensor arrays. Upon skin toxicity, interventions were provided, including scalp cleansing, topical application of corticosteroids for contact dermatitis during array exchange, and anti-infection treatment with topical application of antibiotics.

### 2.4. Treatment compliance and quality of life

Treatment compliance was evaluated monthly through the data on the use of the NovaTTFields-200A device and calculated as a percentage of the daily TTFields usage. The quality of life questionnaire-core 30 (QLQ-C30) (23) and QLQ-brain cancer module (QLQ-BN20) questionnaire (24), provided by the European Organisation for Research and Treatment of Cancer (EORTC), were used to evaluate the health-related quality of life (HRQoL) every 1–3 months. The change in score <10 was defined as stable HRQoL, or else, a decline or improvement was considered.

### 2.5. Statistical analysis

The patient baseline and AEs were obtained by direct counting, and the measured data that did not conform to normal distribution were expressed as the median. The χ^2^ test or Fisher exact test was used for comparison. Data processing was performed using GraphPad Prism 8 (GraphPad Software, La Jolla, CA, USA) and SPSS 23.0 (IBM Corporation, Armonk, New York, USA) software. The starting point of PFS and OS in patients with ndGBM was the time of the first operation, and the starting point of PFS and OS in patients with rGBM was the time of recurrence. The median PFS and OS were analyzed using the Kaplan–Meier survival curves. Multivariate analysis affecting PFS and OS was conducted using the Cox proportional hazards model. Treatment compliance of each patient was expressed in percentage (mean). Comparison of independent datasets between two groups was through the *t*-test, while that among more than two groups was through the one-way ANOVA–Bonferroni multi-comparison test. Statistical significance was set at *P* < 0.05.

## 3. Results

### 3.1. Patient clinical data

In our analysis, a total of 93 patients with GBM were enrolled between January 2013 and May 2021, including 52 ndGBM (55.9%) and 41 rGBM cases (44.1%). Of the 52 ndGBM cases, 13 patients were in the with TTFields group, including seven men and six women with an average age of 54 years (range 33–63 years). Gross total resection (GTR) was performed in six patients, subtotal resection (STR) in seven patients. All patients were IDH wild type; three patients showed the methylation of the MGMT promoter, whereas 10 were unmethylated. Among the 39 patients in the without TTFields group, 24 were men and 15 were women, with an average age of 48 years (range 22–75 years). GTR was performed in 23 patients and STR in 16 patients. All patients were IDH wild type; 13 patients showed MGMT promoter methylation, whereas 26 were unmethylated. No significant differences were noted in gender, age, degree of surgical resection, or MGMT promoter status between the two groups (*P* > 0.05).

Of the 41 rGBM cases, 13 patients were in the with TTFields group, including eight men and five women with an average age of 51 years (range 27–68 years). All patients were IDH wild type; four patients showed the methylation of the MGMT promoter, whereas nine were unmethylated. Among the 28 patients without the TTFields group, 15 were men and 13 were women with an average age of 45 years (range 26–68 years). A total of 14 patients showed MGMT promoter methylation, whereas 14 were unmethylated. No significant differences were noted in gender, age, degree of surgical resection, number of recurrences, or MGMT promoter status between the two groups (*P* > 0.05). Table 1 lists the clinical characteristics of ndGBM and rGBM cases.

**Table 1:** Clinical characteristics of GBM patients.

**Characteristics**	**Newly diagnosed GBM (*****n*** = **52)**			**Recurrent GBM (*****n*** = **41)**		

	**With TTFields (*****n*** = **13)**	**Without TTFields** **(*****n*** = **39)**	***P***	**With TTFields** **(*****n*** = **13)**	**Without TTFields** **(*****n*** = **28)**	***P***
**Median age (year)**						
≤50	3 (23.1%)	19 (48.7%)	0.105	4 (30.8%)	18 (64.3%)	0.091
>50	10 (76.9%)	20 (51.3%)		9 (69.2%)	10 (35.7%)	
**Sex**						
Male	7 (53.8%)	24 (61.5%)	0.624	8 (61.5%)	15 (53.6%)	0.632
Female	6 (46.2%)	15 (38.5)		5 (38.5%)	13 (46.4%)	
**KPS**						
≤70	4 (30.8%)	9 (23.1%)		8 (61.5%)	16 (57.1%)	
>70	9 (69.2%)	30 (76.9%)	0.579	5 (38.5%)	12 (42.9%)	0.79
**Tumor location**						
FL/TL/PL/OL	8 (61.5%)	35 (89.7%)	0.159	9 (69.2%)	23 (82.1%)	0.374
Corpus callosum	4 (30.8%)	2 (5.1%)		3 (23.1%)	2 (7.1%)	
Others	1 (7.7%)	2 (5.1%)		1 (7.7%)	3 (10.7%)	
**Extent of surgery**						
GTR	6 (46.2%)	23 (59.0%)	0.42			
STR	7 (53.8%)	16 (41.0%)				
**MGMT methylation status**						
Methylated	3 (23.1%)	13 (33.3%)	0.729	4 (30.8%)	14 (50.0%)	0.248
Unmethylated	10 (76.9%)	26 (66.7%)		9 (69.2%)	14 (50.0%)	
**Combination therapy**						
TMZ	10 (76.9%)	31 (79.5%)	1	3 (23.1%)	2 (7.1%)	0.348
TMZ + targeted therapy	3 (23.1%)	8 (20.5%)		10 (76.9%)	26 (92.9%)	
**Number of recurrence**						
1st recurrence				6 (46.2%)	20 (71.4%)	0.118
≥2nd recurrence				7 (53.8%)	8 (28.6%)	
**Re-operation**						
GTR				3 (23.1%)	8 (28.6%)	0.362
STR				2 (15.4%)	9 (32.1%)	
No				8 (61.5%)	11 (39.3%)	

GBM, glioblastoma; TTFields, tumor-treating fields; KPS, Karnofsky performance status; FL, frontal lobe; TL, temporal lobe; PL, parietal lobe; OL, occipital lobe; GTR, gross total resection; STR, subtotal resection; MGMT, O6-methylguanine-DNA methyltransferase; TMZ, temozolomide.

### 3.2. Survival outcome

**Figure 1:**
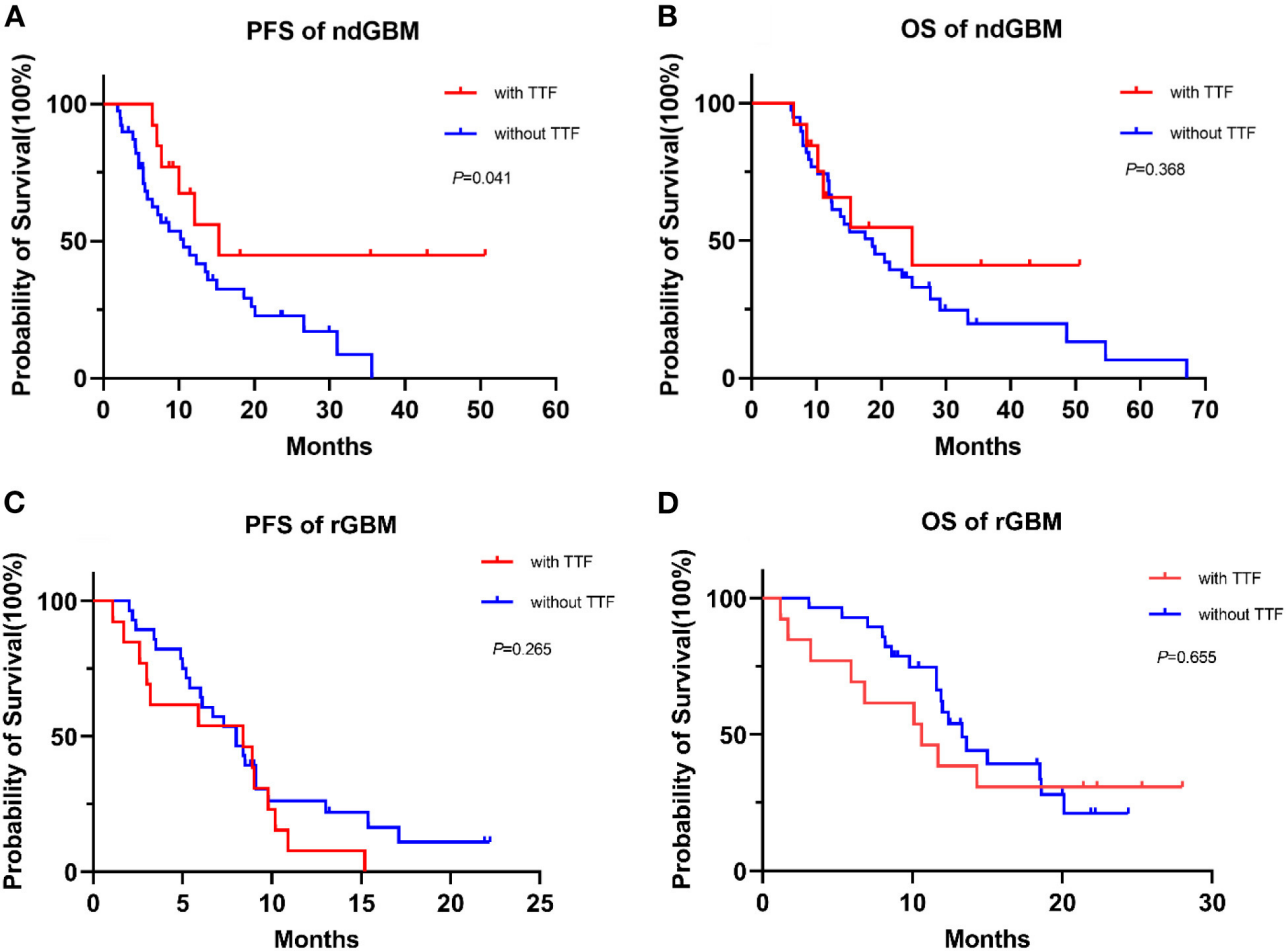
The survival analysis of the newly diagnosed glioblastoma (ndGBM) and recurrent GBM (rGBM) in two groups. **(A)** Progression-free survival (PFS) of ndGBM; **(B)** overall survival (OS) of ndGBM; **(C)** PFS of rGBM; **(D)** OS of rGBM.

The follow-up period was 34.7 months (95% CI: 26.5–42.9) in the ndGBM cohort. Among the patients with ndGBM, there were 13 cases with TTFields and 39 with no TTFields. The median PFS was 15.3 months (95% CI: 6.5–24.1) and 10.6 months (95% CI: 5.4–15.8) in the two groups, respectively, with *P* = 0.041. The 1-year PFS rate was 67.3 and 44.8% in the two groups, respectively. The median OS was 24.8 months (95% CI: 6.8–42.8) and 18.6 months (95% CI: 1.4–25.8), respectively, with *P* = 0.368. The 1-year OS rate was 65.8 and 66.7% in the two groups, respectively (Figures 1A, B).

**Figure 2:**
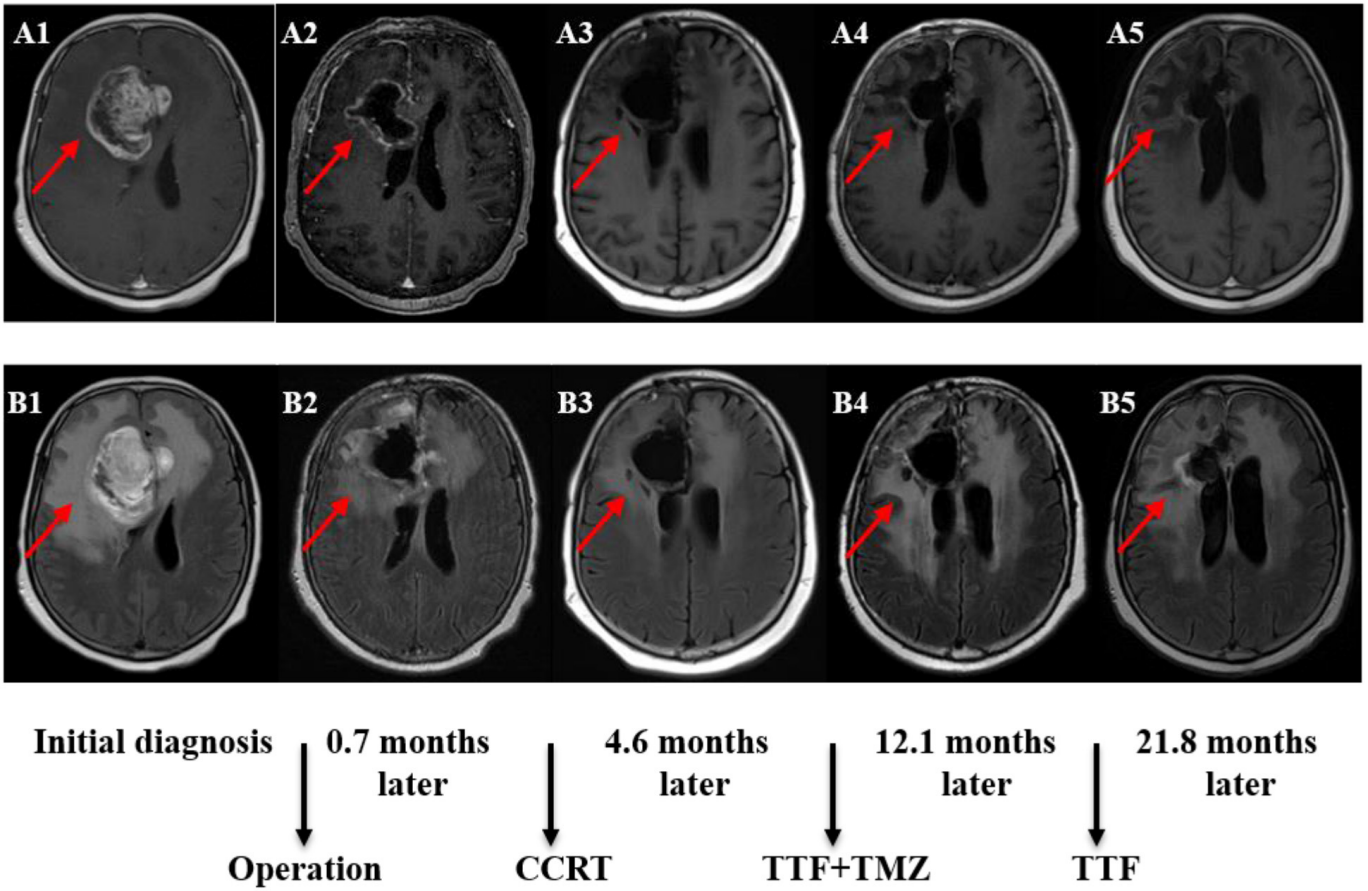
MRI (Magnetic Resonance Imaging) changes of newly diagnosed glioblastoma (GBM). **(A1–A5)** Contrast-enhanced MRI; **(B1–B5)** MRI-Flair. MRI images followed-up every 2 months before and after treatment. Only some of the images were exhibited. The arrow indicates the tumor and tumor bed. This patient has followed-up for 35.4 months with a stable disease.

**Figure 3:**
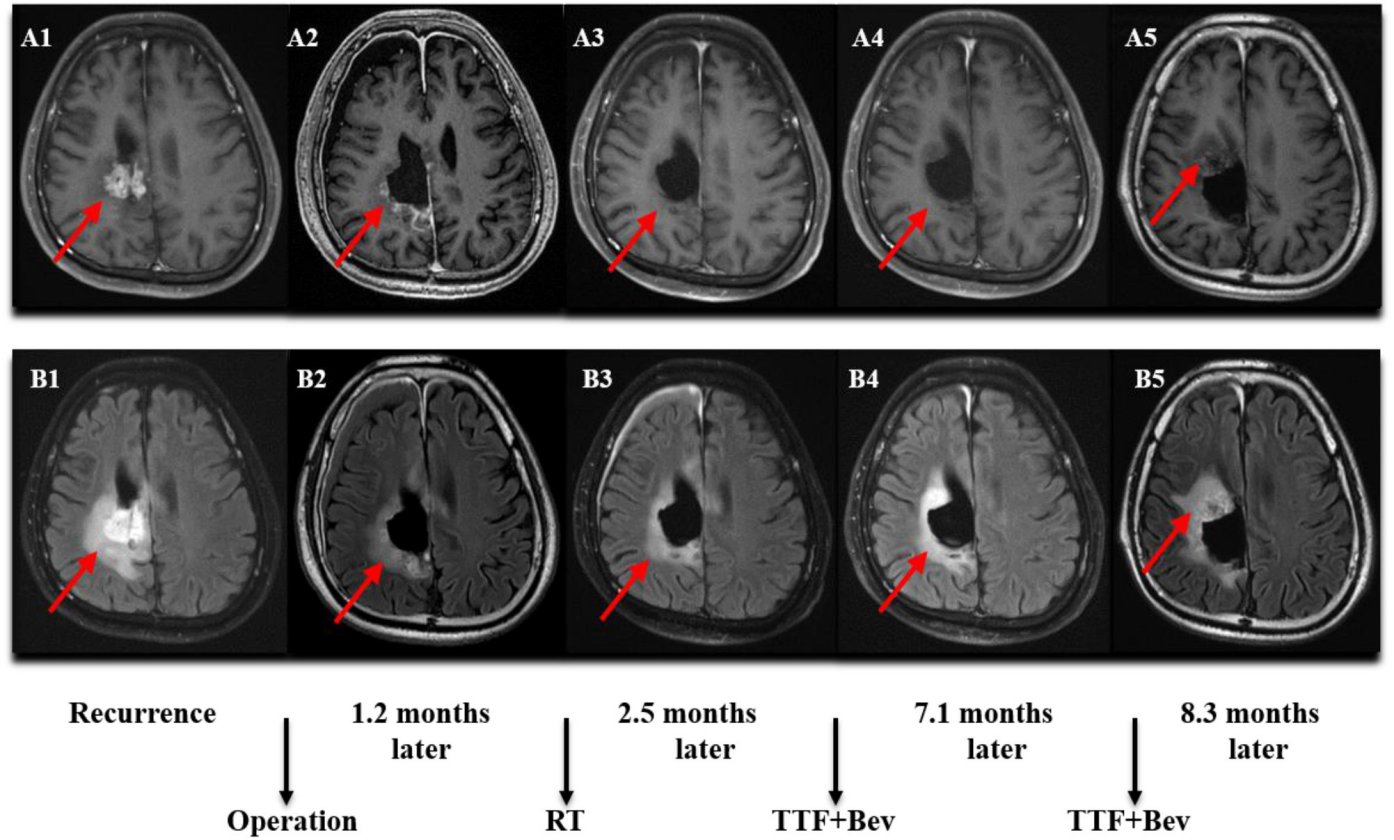
MRI (Magnetic Resonance Imaging) changes of recurrent GBM (rGBM). **(A1–A5)** Contrast-enhanced MRI; **(B1–B5)** MRI-Flair. MRI images followed-up every 2 months before and after treatment. Only some of the images were exhibited. The arrow indicates the tumor and tumor bed. This patient was followed-up for 8.3 months and he developed a disease progression.

The follow-up period was 21.8 months (95% CI: 20.6–23.1) in the rGBM cohort. Among the patients with rGBM, there were 13 cases with TTFields and 28 with no TTFields. The median PFS was 8.4 months (95% CI: 1.7–15.2) and 8.0 months (95% CI: 5.8–10.2) in the two groups, respectively, with *P* = 0.265. The 1-year PFS rate was 7.7 and 26.2% in the two groups, respectively. The median OS was 10.6 months (95% CI: 4.8–16.4) and 13.3 months (95% CI: 11.0–15.6), respectively, with *P* = 0.655. The 1-year OS rate was 38.5 and 62.2% in the two groups, respectively (Figures 1C, D). Dynamic changes in the MRI scans of representative ndGBM and rGBM cases are shown in Figures 2, 3.

Among the patients with ndGBM, the patients with female (*P* = 0.026), KPS > 70 (*P* < 0.001), GTR (*P* < 0.001), and TTFields (*P* = 0.041) had better PFS. The patients with KPS > 70 and MGMT methylation had better OS. A multivariate analysis showed that KPS > 70 (*P* < 0.001; HR 0.181, 95% CI: 0.072–0.456) and GTR (*P* = 0.001; HR 0.23, 95% CI: 0.1–0.527) were favorable independent prognostic factors for PFS in patients with ndGBM. KPS > 70 (*P* = 0.003; HR 0.247, 95% CI: 0.099–0.616) and MGMT methylation (*P* = 0.004; HR 3.443, 95% CI: 1.484–7.987) were favorable independent prognostic factors for OS (Table 2).

**Table 2:** Univariate and multivariate analysis for PFS and OS of ndGBM.

**Variable**	**PFS**			**OS**		
	**Univariate analysis**	**Multivariate analysis**		**Univariate analysis**	**Multivariate analysis**	
	***P*** **-value (log-rank)**	**Hazard ratio (95% CI)**	***P*** **-value**	***P*** **-value (log-rank)**	**Hazard ratio (95% CI)**	***P*** **-value**
Age (years): ≤50 vs. >50	0.476			0.172		
Sex: female vs. male	**0.026**	1.427 (0.978–2.082)	0.065	0.071	1.164 (0.796–1.704)	0.434
KPS: >70 vs. ≤70	**<0.001**	0.181 (0.072–0.456)	**<0.001**	**0.014**	0.247 (0.099–0.616)	**0.003**
Extent of surgery: GTR vs. STR	**<0.001**	0.23 (0.1–0.527)	**0.001**	0.088	0.498 (0.246–1.007)	0.052
MGMT: meth vs. unmeth	0.122			**0.02**	3.443 (1.484–7.987)	**0.004**
TTFields: with vs. without	**0.041**	0.609 (0.203–1.825)	0.375	0.371	1.21 (0.445–3.286)	0.709

PFS, progression-free survival; OS, overall survival; KPS, Karnofsky performance status; GTR, gross total resection; STR, subtotal resection; MGMT, O6-methylguanine-DNA methyltransferase; 95% CI, 95% confidence interval; TTFields, tumor-treating fields. The bold values mean the difference is statistically significant.

Among the patients with rGBM, a single-factor analysis showed that females (*P* = 0.02), KPS > 70 (*P* = 0.012), re-operation (STR + GTR) (*P* = 0.002), and first recurrence (*P* = 0.027) had better PFS. The patients with KPS > 70 (*P* = 0.001), re-operation (*P* = 0.003), and first recurrence (*P* = 0.003) had better OS. The multivariate analysis also confirmed that females (*P* = 0.012; HR 2.785, 95% CI: 1.25–6.203), re-operation (*P* < 0.001; HR 4.23, 95% CI: 2.026–8.834), and first recurrence (*P* = 0.032; HR 0.434, 95% CI: 0.203–0.931) were favorable independent prognostic factors for PFS. KPS > 70 (*P* = 0.022; HR 3.778, 95% CI: 1.211–11.787), re-operation (*P* = 0.019; HR 3.125, 95% CI: 1.207–8.235), and first recurrence (*P* < 0.001; HR 0.148, 95% CI: 0.057–0.387) were favorable independent prognostic factors for OS (Table 3).

**Table 3:** Univariate and multivariate analysis for PFS and OS of rGBM.

**Variable**	**PFS**			**OS**		
	**Univariate analysis**	**Multivariate analysis**		**Univariate analysis**	**Multivariate analysis**	
	***P*** **-value (log-Rank)**	**Hazard ratio (95% CI)**	***P*** **-value**	***P*** **-value (log-rank)**	**Hazard ratio (95% CI)**	***P*** **-value**
Age (years): ≤50 vs. >50	0.893			0.814		
Sex: female vs. male	**0.02**	2.785 (1.25–6.203)	**0.012**	0.204		
KPS: >70 vs. ≤70	**0.012**	1.723 (0.727–4.082)	0.217	**0.001**	3.778 (1.211–11.787)	**0.022**
Re-operation: no vs. STR + GTR	**0.002**	4.23 (2.026–8.834)	**<0.001**	**0.003**	3.152 (1.207–8.235)	**0.019**
MGMT: meth vs. unmeth	0.262			0.173		
TTFields: with vs. without	0.265			0.655		
Number of recurrence: 1st vs. ≥2nd	**0.027**	0.434 (0.203–0.931)	**0.032**	**0.003**	0.148 (0.057–0.387)	**<0.001**

PFS, progression-free survival; OS, overall survival; KPS, Karnofsky performance status; MGMT, O6-methylguanine-DNA methyltransferase; 95% CI, 95% confidence interval; TTFields, tumor-treating fields. The bold values mean the difference is statistically significant.

Through the subgroup analysis of patients with ndGBM, we found that in patients without TTFields, the PFS of patients with GTR was significantly better than that of patients with STR (median survival:19.6 vs. 5.3 months; *P* < 0.001). Among the patients using TTFields, there was no significant difference in PFS between GTR and STR (*P* = 0.518). However, we also found that patients with STR who used TTFields had better PFS than those who did not (*P* = 0.003). Among the patients who did not use TTFields, the OS of patients with GTR was significantly better than that of patients with STR (median survival: 24.8 vs. 13.7 months; *P* = 0.008). Among the patients using TTFields, there was no significant difference in OS between total and subtotal resection (*P* = 0.403).

### 3.3. Toxicity, treatment compliance, and quality of life

**Figure 4:**
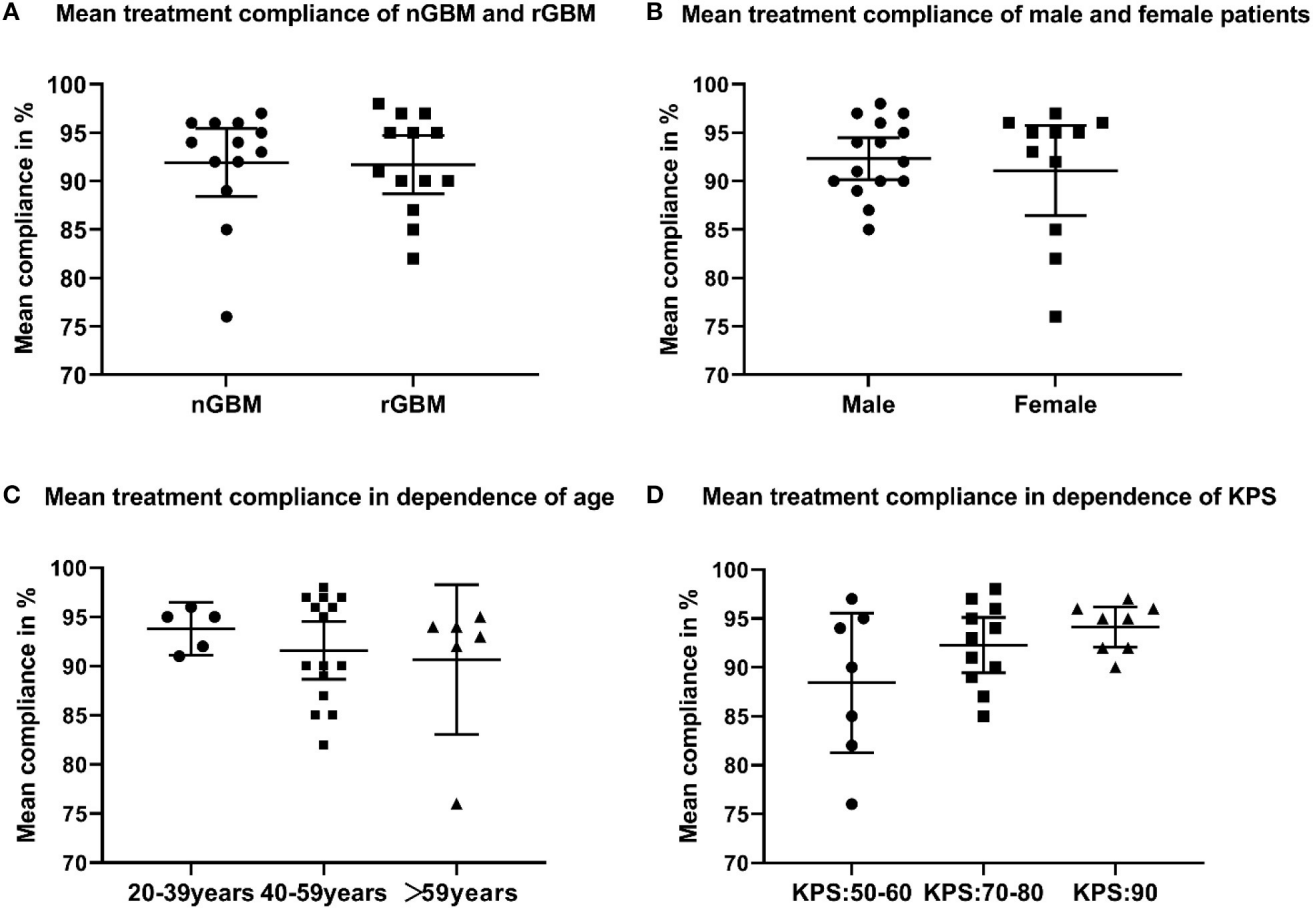
**(A)** Mean treatment compliance of newly diagnosed and recurrent glioblastoma (GBM); **(B)** mean treatment compliance of male and female patients who received TTFields; **(C)** mean treatment compliance in dependence of age; **(D)** mean treatment compliance in dependence of Karnofsky performance score.

Among all patients treated with an electric field, 21 cases had dAE (21/26, 80.8%), including 17 cases of grade 1, three cases of grade 2, and one case of grade 3. Common dAEs were dermatitis, ulcers, and bursitis. All the dAEs could be resolved or controlled by the topical application of glucocorticoids or antibiotics. The average treatment compliance rate was 91.9% in ndGBM cases vs. 91.7% in rGBM cases (*P* = 0.90, Figure 4A), while 92.3% in men vs. 91.3% in women (*P* = 0.21, Figure 4B). Based on the different age groups, the treatment compliance rate was 93.8% in 20–39-year individuals vs. 91.6% in 40–59-year individuals vs. 90.7% in the>59 years old group (*P* = 0.62, Figure 4C). In addition, according to the preoperative KPS scores, the treatment compliance rate in patients with KPS scores of 50–60, 70–80, and 90 was 88.4, 92.3, and 94.1%, respectively, with no statistically significant differences (*P* = 0.11, Figure 4D). A total of 22 cases showed a stable HRQoL, two showed improvement, manifested in cognitive and social functioning, and two showed a decline, mainly in emotional and role functioning.

### 3.4. Biomarkers of patients with TTFields

Gene detection in patients with GBM treated with an electric field is shown in Table 4. We explored the relationship between some genes and survival and found no statistically significant correlation. However, of the 13 ndGBM cases, four cases with BRAF-V600E mutations did not show recurrence during follow-up. In addition, three cases with amplification in the EGFR gene showed worse PFS. Of the six patients with rGBM showing the first recurrence, two with the activation of proangiogenic pathways, including amplifications in KIT, FGFR, PDGFR, or KDR genes, showed the longest PFS.

**Table 4:** The gene detection of TTFields group.

**Gene detection**	**Newly diagnosed GBM (*n* = 13)**	**Recurrent GBM (*n* = 13)**
	**No. of patients (%)**	**No. of patients (%)**
BRAF-V600E mutation	4 (30.8)	
PI3K mutation	3 (23.1)	3 (23.1)
EGFR amplification	3 (23.1)	2 (15.4)
KIT, FGFR, PDGFR, or KDR amplification	2 (15.4)	3 (23.1)
TERT mutation	6 (46.2)	8 (61.5)
P53 mutation	2 (15.4)	4 (30.8)
PTEN mutation	2 (15.4)	
CDKN2A/2B co-deletion	1 (7.7)	
FGFR3-TACC3 refusion		1 (7.7)
MET amplification		1 (7.7)
DDR1 mutation		1 (7.7)
CK4 amplification	1 (7.7)	

GBM, glioblastoma; TTFields, tumor-treating fields.

## 4. Discussion

The findings of this study showed that the median compliance rates among the ndGBM and rGBM cases for TTFields therapy were 94 and 91%, respectively. TTFields therapy was performed for an average of 18 h daily (100%) in all patients. This could be attributed to careful education before treatment, family support, close monitoring during treatment, and timely management of toxicity. The *post-hoc* analysis of the EF-14 study suggests the necessity for the continuous use of the TTFields device as the treatment efficacy was found to be positively associated with patient compliance. It was proven that the duration of TTFields up to 18 h daily, with a treatment compliance of >75%, could lead to significant therapeutic outcomes. A duration >22 h daily is reportedly associated with a 29.3% survival rate in 5 years (25). Based on our findings, we found that high treatment compliance was independent of age, gender, preoperative KPS score, and the stage of disease of the patients. This implied that elderly patients or those with a poor quality of life could also undergo TTFields. In our cohort, two patients accepted the TTFields therapy for over 19 months and continue to use it with good compliance. The long-term use of TTFields appeared to show no substantial effects on patient compliance. In the EF-11 study, the median compliance rate of patients was 86%, and in the EF-14 study, <10% of patients showed a compliance rate of 90% (18, 20). In our study, the compliance rate was markedly higher, which may have contributed to the more favorable survival outcomes.

In the ndGBM group in our study, the median PFS of patients with TTFields was better than that of patients without TTFields (15.3 vs. 10.6 months; *P* = 0.041). This result was similar to the EF-14 study. However, the 1-year PFS rate of the TTFields group was 67.3%, markedly better than the results reported in the EF-14 study (1-year PFS <40%). In detail, 90% compliance in the EF-14 study was <10%, while according to our findings, it was 76.9%. This may be accounting for the superior PFS in our study. The median OS was 24.8 months (95% CI: 6.8–42.8) and 18.6 months (95% CI: 11.4–25.8), respectively, with *P* = 0.368. The median OS of the two groups in our study was comparable, which might be attributed to our patients' continued active therapy following recurrence.

It has been reported that in ndGBM, the survival of patients with GTR is significantly better than that of patients with STR (26, 27). Our study also found that GTR (*P* = 0.001; HR 0.23, 95% CI: 0.1–0.527) was a favorable independent prognostic factor for PFS. Among patients without TTFields, patients with GTR had significantly better PFS and OS than patients with STR (*P* < 0.001; *P* = 0.008). This is consistent with data in the literature (28). However, there was no significant difference between PFS in patients with GTR and STR in patients with TTFields (*P* = 0.518). Therefore, a subgroup analysis was performed and found that in the STR group, patients with TTFields had better PFS than those without TTFields (*P* = 0.003). This may be because TTFields improved the survival of patients with STR, thereby narrowing the survival gap between patients with GTR and STR in the TTFields group. By univariate analysis, we found that KPS > 70 was also an independent prognostic factor for PFS and OS. MGMT methylation is a favorable independent prognostic factor for OS. The results were similar to the findings in other studies (29, 30).

There is no consensus on whether mutations in BRAF-V600E are associated with a better prognosis, but several studies support that EGFR amplification is a significant risk factor for poor survival outcomes (31–34). In our study, four with BRAF-V600E mutations showed better PFS. Contrastingly, three cases of ndGBM with EGFR amplification exhibited worse survival outcomes. Due to the small sample size, we cannot determine whether BRAF-V600E and EGFR are biomarkers of favorable outcomes from TTFields, and subsequent studies with large samples are needed to further determine.

In the rGBM group in our study, the median PFS and OS data showed no significant difference between the two groups with and without TTFields, which was consistent with the findings of the EF-11 study. The 6-month PFS and 1-year OS rates of the TTFields group were 53.8 and 38.5%, respectively. While in the EF-11 study, the 6-month PFS in rGBM cases who underwent TTFields therapy was 21.4%. As compared to the EF-11 study, patients with rGBM in our study showed higher survival rates. Several possible reasons may account for it. First, in the EF-11 study, all patients underwent TTFields therapy alone. In our study, all rGBM cases received TTFields combination therapy, including re-operation, targeted therapy, or TMZ-based chemotherapy. Many studies confirm that combining TTFields and other anti-tumor therapies (such as radiotherapy, chemotherapy, and immunotherapy) yield better therapeutic outcomes (35–38). In addition, in our cohort, 46.2% of patients showed a first recurrence, significantly higher than the 9% in the EF-11. The median compliance of patients with rGBM in our study (91%) was also higher than that in the EF-11 study (86%).

Univariate and multivariate analyses of rGBM showed that gender, KPS, re-operation, and a number of recurrences were significant prognostic factors for PFS, while KPS, re-operation, and a number of recurrences were significant prognostic factors for OS. This was similar to the results of other previous studies (39, 40). Several treatment regimens for rGBM were used in our study, so the results demonstrate that using TTFields was not a prognostic factor for survival. In follow-up studies, a more rigorous research protocol should be developed to remove the influence of confounding factors and to draw more reliable conclusions.

In the six rGBM cases with the first recurrence, five cases underwent re-operation. As evidenced by sequencing the tumor samples acquired after re-operation, we found two cases with the activation of proangiogenic pathways, including amplifications in KIT, FGFR, PDGFR, or KDR, and they exhibited the longest PFS. None of these gene amplifications are known to be associated with favorable survival outcomes (41). This suggested that the rGBM cases with active angiogenic signaling might benefit more from the TTFields therapy. A previous study reports that PTEN mutations predict benefits from TTFields therapy in patients with rGBM (42). However, in our study, no PTEN mutations were identified in the rGBM group. This may be attributed to the tumor samples used for sequencing in their study, which were acquired from initial surgery; genetic alterations occur over time, and with treatment intervention, the genetic characteristics in rGBM may differ from those after primary resection (43, 44).

Moreover, we also used the QLQ-C30 and QLQ-BN20 questionnaires to assess the safety of the TTFields therapy. Of the 26 cases, 22 showed a stable HRQoL and two exhibited improvements, which mainly manifested in cognitive and social functioning. This was consistent with the findings of a previous report (45). The common AE was dAEs in 21 cases (21/26, 80.8%), a little higher than for the Korean patients reported in the EF-14 study.

The current study has some limitations. This was a single-center study, and potential biases may exist in patient selection. The sample size was relatively small. A large sample size and prospective control trials are needed in future.

## 5. Conclusion

In conclusion, TTFields showed good efficacy in ndGBM, especially in patients with STR. However, TTFields failed to improve the survival of rGBM. In addition, this treatment is safe and tolerable. A larger sample size and randomized controlled clinical trials are needed to further verify the effectiveness of TTFields treatment.

## Data availability statement

The original contributions presented in the study are included in the article/supplementary material, further inquiries can be directed to the corresponding authors.

## Ethics statement

The studies involving human participants were reviewed and approved by the Ethics Committee of Xiangya Hospital of Central South University. Written informed consent for participation was not required for this study in accordance with the national legislation and the institutional requirements.

## Author contributions

CL: conceptualization and supervision. LSh, LSu, XG, and CL: data curation. LSh: formal analysis and methodology. XG and CL: funding acquisition. LSu and CL: investigation. LSh and LSu: visualization. LSh and XG: writing the original draft. LSh, XG, and CL: writing, reviewing, and editing. All authors have read and agreed to the published version of the manuscript.
